# Rethinking biliary reconstruction: alternatives to standard Roux-en-Y hepaticojejunostomy

**DOI:** 10.1093/jscr/rjaf542

**Published:** 2025-07-21

**Authors:** Omar Barakat, Samuel P Creden, David M Holmes, Waleed Ageedi

**Affiliations:** Department of Surgery, Division of Surgical Oncology and Hepatobiliary and Pancreas Surgery, Baylor College of Medicine, 7200 Cambridge St., 7th Floor, Houston, TX 77030, United States; Department of Surgery, Baylor College of Medicine, One Baylor Plaza, Houston, TX 77030, United States; Department of Surgery, Baylor College of Medicine, One Baylor Plaza, Houston, TX 77030, United States; Department of Surgery, Baylor College of Medicine, One Baylor Plaza, Houston, TX 77030, United States

**Keywords:** bile duct obstruction, Roux-en-Y anastomosis, biliary tract surgical procedures, hepaticojejunostomy

## Abstract

Roux-en-Y hepaticojejunostomy enables tension-free bile-duct reconstruction and minimizes reflux. However, factors like a short mesentery or severe peri-hilar inflammation may prevent the jejunal loop from reaching the hepatic hilum, making safe anastomosis unfeasible. We have developed a novel surgical technique that overcomes these challenges. In this case report, we describe our triple bypass technique, in which a vascularized gastric tube graft is anastomosed to the common bile duct, and we present short-term outcomes from the use of this technique in a patient with chronic pancreatitis; overall, this patient recovered well, with a viable graft and no leakage. This novel technique expands surgical options for complex extrahepatic bile-duct reconstruction.

## Introduction

Roux-en-Y hepaticojejunostomy is the gold standard for bile-duct reconstruction after bile-duct injury or after resection for benign or malignant bile-duct obstruction. Roux-en-Y hepaticojejunostomy provides tension-free anastomosis and minimizes bile and intestinal reflux [1–3].

This technique has proven highly effective in restoring bile-duct continuity and managing complex biliary injuries. However, the jejunal loop occasionally does not reach the hepatic hilum due to a short mesentery or severe peri-hepatic and peri-colonic inflammation, impeding a safe, tension-free anastomosis.

In 2023, we reported a novel technique that uses a peritoneal tubular graft to construct the bile duct [4]. Here, we describe another novel surgical technique aimed at overcoming difficulties encountered when using the Roux-en-Y approach to reconstruct the bile duct. The procedure adheres to the ethical standards of Baylor College of Medicine and the Helsinki Declaration of the World Medical Association.

## Case presentation

**Figure 1:**
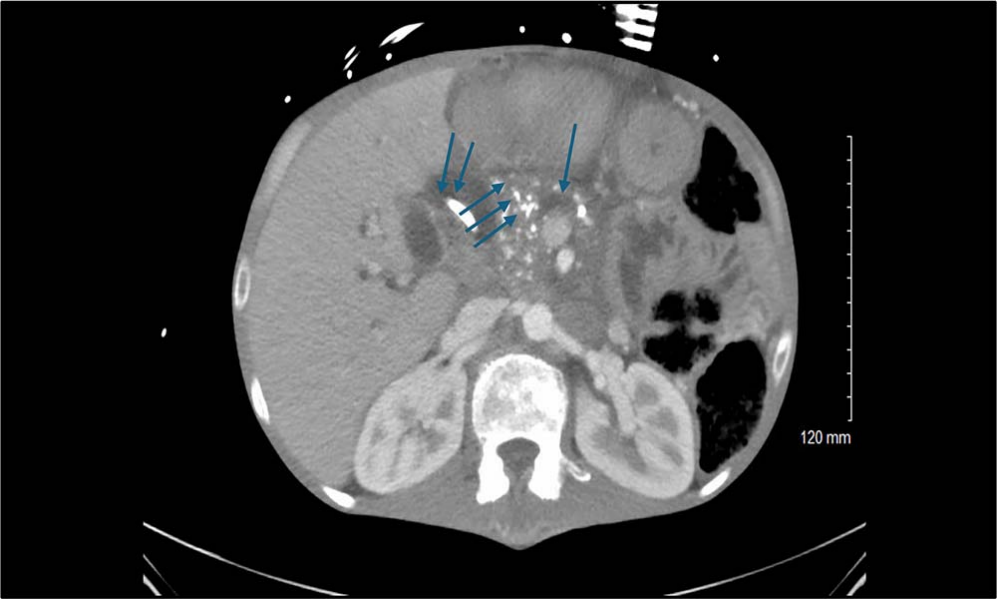
Preoperative computed tomography scan of the abdomen showing the calcified head of the pancreas (triple arrows), dilated pancreatic duct (single arrow), and dilated common bile duct (double arrows).

A 44-year-old man with a history of severe protein-calorie malnutrition (body mass index, 16), chronic hyponatremia, type 2 diabetes, and alcohol-induced chronic pancreatitis presented to our unit for surgical evaluation. Baseline laboratory findings included a total bilirubin of 4 mg/dl, a markedly elevated alkaline phosphatase level of 2848 IU/L, and clinical and biological evidence of severe malnutrition (Table 1). Abdominal computed tomography revealed common bile-duct dilatation (2.1 cm), scattered pancreatic parenchymal calcifications, main pancreatic duct dilatation (5 mm), duodenal strictures, and extensive peri-pancreatic inflammatory changes encasing the pancreatic head and superior mesenteric vein/portal vein, without vascular occlusion (Fig. 1). The biliary stricture had led to both intrahepatic and extrahepatic ductal dilatation. Endoscopic stenting was not feasible due to the duodenal stricture, necessitating insertion of a percutaneous transhepatic biliary drainage catheter.

**Table 1:** Preoperative and postoperative liver function test results

**Test (normal range)**	**Preoperative**	**Postoperative**
Total bilirubin, mg/dl (0.0–1.2)	4.0	0.2
AST, U/L (5–34)	96	10
ALT, U/L (6–55)	109	12
Alkaline phosphatase, IU/L (44–121)	2884	185
Albumin, mg/dl (3.7–4.7)	2.7	3.1
Prealbumin, mg/dl (14–37)	10	10

Abbreviations: ALT, alanine transaminase; AST, aspartate aminotransferase.

Given the extensive inflammation and vascular involvement, pancreaticoduodenectomy was not feasible. Instead, a triple bypass involving the pancreas, bile duct, stomach, and a Roux-en-Y loop of jejunum was planned. The patient underwent 3 weeks of aggressive preoperative nutritional optimization via total parenteral nutrition.

## Operative procedure and findings

At laparotomy, dense adhesions were lysed, and the gallbladder was removed in a retrograde fashion. The common bile duct was noted to be significantly dilated (3 cm). Hilar dissection was limited by severe inflammation and anatomical distortion.

The duodenum was mobilized. The lesser sac was entered, and the posterior wall of the stomach was dissected away from the anterior capsule of the pancreas. The main pancreatic duct was palpated, cannulated, incised longitudinally, and examined by using a choledochoscope. The incision was extended further toward the tail and neck of the pancreas to release multiple intra-ductal strictures and to extract multiple stones.

The proximal jejunum was divided 35 cm from the duodenojejunal junction, and the distal limb was brought through the transverse mesocolon. Interrupted 3–0 silk and 5–0 synthetic polypropylene sutures were used to construct a two-layer, side-to-side pancreatojejunostomy (PJ).

Due to severe inflammation and fibrosis surrounding the duodenum and hepatoduodenal ligament, a hepaticojejunostomy downstream to the PJ could not be safely performed. Therefore, a vascularized gastric tubular graft was fashioned. The greater curvature of the pyloric antrum was stapled ~2 cm from the edge and extended in a cephalad direction, creating a 6-cm gastric conduit while preserving both the right and left gastroepiploic arcades. The conduit was isolated from the stomach at its proximal end by using an Endo-GIA stapler (Covidien, North Haven CT, USA). After adequate perfusion from the left gastroepiploic arcade was confirmed, the right gastroepiploic artery was ligated and divided to allow free conduit mobilization.

The anterior surface of the common bile duct was incised transversely. An end-to-side anastomosis between the common bile duct and the gastric conduit was created by using interrupted 5–0 polydioxanone (PDS) sutures. The distal end of the conduit was then anastomosed end-to-side to the PJ loop in two layers by using 3–0 silk and 4–0 PDS sutures.

**Figure 2:**
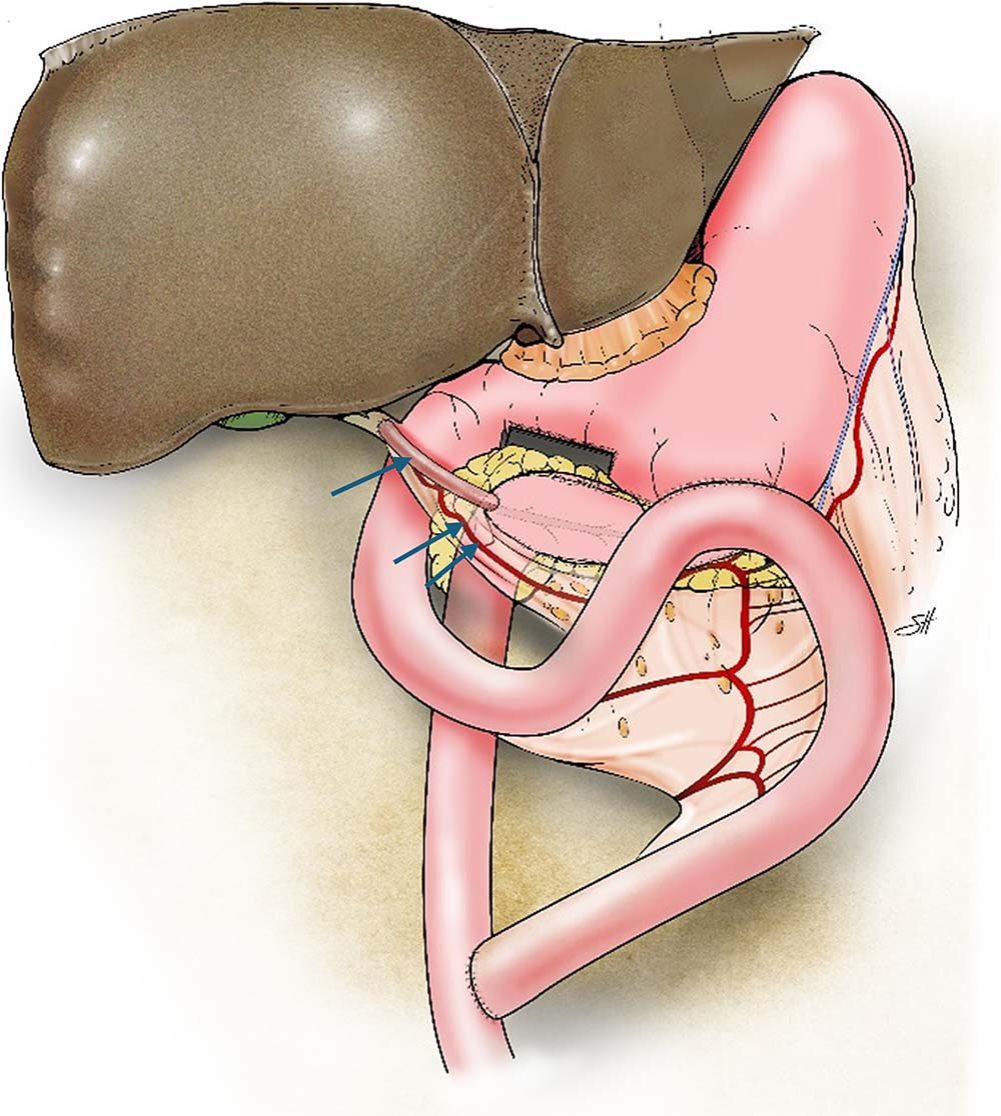
Illustration of the triple bypass depicting reconstruction of the Roux-en-Y PJ, loop gastrojejunostomy, and the tubular gastric conduit (single arrow) created from the greater curvature of the stomach while maintaining the left gastroepiploic arcade (double arrows) to complete the end-to-side hepaticojejunostomy. Copyright Baylor College of Medicine.

Finally, the proximal jejunal limb was used to construct side-to-side gastrojejunostomy on the posterior wall of the stomach by using the Endo-GIA stapling device (Covidien). The jejunojejunostomy was constructed 50 cm distal to the biliary anastomosis (Fig. 2).

## Postoperative course

The patient had an uneventful postoperative course and was discharged on postoperative day 6. At the 6-month follow-up, cholangiography through the percutaneous transhepatic biliary drainage catheter showed a patent biliary conduit without evidence of leakage. Liver function improved overall, with total bilirubin having decreased to 0.2 mg/dl and alkaline phosphatase to 185 IU/L (Table 1).

## Discussion

Successful anastomosis during Roux-en-Y hepaticojejunostomy relies on two key factors: well-vascularized bile-duct mucosa and tension-free mucosa-to-mucosa anastomosis.

Previously, we described our use of a peritoneal tubular graft to reconstruct the bile duct in a patient whose a short mesentery prevented the distal loop of jejunum from reaching the liver hilum for a safe hepaticojejunostomy [4]. We subsequently reported the use of an isolated distal gastric pouch to repair duodenal and bile-duct perforations after endoscopic retrograde cholangiopancreatography [5].

Here, we highlight a novel triple-bypass technique for managing chronic pancreatitis. We utilized the proximal jejunum to bypass the stomach, pancreas, and bile duct. An isolated vascularized tubular conduit was fashioned from the greater curvature of the stomach to facilitate a safe and tension-free hepaticojejunostomy. The gastric conduit, ~6 cm in length, was created while preserving the left gastroepiploic arcade. Dividing the right gastroepiploic artery allowed for the greater reach of the graft without compromising its blood supply. This maneuver was critical for successful tension-free anastomosis to the common bile duct.

The technique was not only technically feasible and safe, but also advantageous in circumstances wherein a standard Roux-en-Y loop could not be mobilized due to surrounding dense inflammation. Constructing an additional Roux-en-Y loop distal to the PJ was considered, but it ultimately was not feasible due to severe restriction in jejunal mobility caused by chronic peripancreatic inflammation.

## Conclusion

This novel technique offers a new option for complex biliary reconstruction, regardless of whether end-to-end anastomosis or bypass to a jejunal limb is required. We believe the technique represents a valuable addition to the surgical armamentarium for both benign and malignant conditions involving the extrahepatic bile duct.
