# Correction to: Rethinking biliary reconstruction: alternatives to standard Roux-en-Y hepaticojejunostomy

**DOI:** 10.1093/jscr/rjag094

**Published:** 2026-04-25

This is a correction to: Omar Barakat, Samuel P Creden, David M Holmes, Waleed Ageedi, Rethinking biliary reconstruction: alternatives to standard Roux-en-Y hepaticojejunostomy, *Journal of Surgical Case Reports*, Volume 2025, Issue 7, July 2025, rjaf542, https://doi.org/10.1093/jscr/rjaf542

The following was added to the caption for Figure 2: “Copyright Baylor College of Medicine.” In the **Acknowledgments** section, the following statement was added: “The authors would like to thank Scott Holmes, CMI, a member of the Michael E. DeBakey Department of Surgery at Baylor College of Medicine, for graphic assistance during the preparation of this manuscript.”

